# A rare cystic and papillary variant of acinar cell carcinoma with liver metastasis and molecular analysis

**DOI:** 10.1007/s00428-025-04315-y

**Published:** 2025-10-22

**Authors:** Meredith K. Herman, Eric Chang, Jiaqi Shi

**Affiliations:** 1 Department of Pathology & Clinical Labs, University of Michigan, 2800 Plymouth Road, Building 35, Ann Arbor, MI 48109, USA

**Keywords:** Pancreatic ductal adenocarcinoma, Intraductal cystic neoplasm, Acinar cystic transformation, Acinar-to-ductal metaplasia, NRAS mutation

## Abstract

Acinar cell carcinoma (ACC) is a pancreatic neoplasm with histologic variants that pose significant diagnostic challenges. We report a 75-year-old man with a previously unreported cystic and papillary ACC metastatic to the liver. Histologically, the tumor exhibited cystic and papillary proliferations of epithelial cells with eosinophilic cytoplasm, prominent nucleoli, and frequent mitoses. Immunohistochemical analysis showed positivity for trypsin and BCL-10, confirming ACC. The cyst lining displayed both acinar and ductal features, suggesting origin from acinar cystic transformation with acinar-to-ductal metaplasia rather than a true intraductal neoplasm. Molecular studies identified NRAS, CTNNB1, and SMAD4 mutations. Despite reports of indolent behavior in similar variants, our patient developed peritoneal metastasis and died 2 years after surgery and chemotherapy, highlighting the potential for aggressive clinical behavior and the importance of distinguishing these tumors from benign cystic pancreatic neoplasms.

## Introduction

Acinar cell carcinoma (ACC) is a rare, aggressive malignancy of the exocrine pancreas which accounts for 1–2% of all pancreatic neoplasms in older adults and pediatric populations, conferring a poor prognosis [1–3]. Classic ACC is often large, ranging 8–10 cm in size, and characterized by scant fibrous stroma and solid hypercellular growth of epithelial neoplastic cells that have a moderate amount of eosinophilic granular cytoplasm, uniform nuclei, and single prominent nucleoli [3]. Multiple architectural patterns have been described. The most common ACC growth patterns include solid, acinar, ductal, and trabecular [4]. Subtypes of ACC include acinar cell cystadenocarcinoma, mixed acinar neuroendocrine carcinoma, and mixed acinar ductal adenocarcinoma, with less common patterns showing oncocytic, spindle, clear, and pleomorphic morphology [1]. Moreover, several documented variants of ACC can be misdiagnosed as intraductal neoplasms, including intraductal papillary mucinous neoplasm (IPMN) [5], intraductal oncocytic papillary neoplasm (IOPN) [6], and intraductal tubulopapillary neoplasm (ITPN) [7], with or without associated invasive adenocarcinoma. These variants can also mimic pancreatic ductal adenocarcinoma with cystic papillary features [8], solid pseudopapillary neoplasm (SPN) [9], pancreatic neuroendocrine tumors (PNETs) [10], and pancreatoblastoma (PB) [11]. Although careful histologic evaluation is paramount, molecular studies may offer crucial insights to resolve diagnostic ambiguities [12].

Herein, we report a diagnostically challenging and novel case of acinar cell carcinoma exhibiting extensive cystic and papillary architectural features with similar morphology in the liver metastasis, a presentation that has not previously been reported. This paper aims to contribute to the literature by describing this extremely rare cystic and papillary variant of ACC metastasized to the liver and addressing differential diagnoses.

## Case report

### Patient presentation

A 75-year-old male with a past medical history of hypertension and hyperlipidemia initially presented with 3 days of worsening epigastric pain, bloating, nausea, vomiting, fever, and chills. The patient also reported 2 years of slow progressive weight loss, which had acutely worsened over the past 2 months, with unintentional weight loss of nearly 30 lbs. During his admission, his labs were significant for mild leukocytosis with a WBC of 10.3 (K/uL) and normal liver tests (aspartate aminotransferase (AST): 21 U/L; alanine aminotransferase (ALT): 17 U/L; alkaline phosphatase (ALP): 93 U/L; total bilirubin 0.7 mg/dL; and direct bilirubin 0.3 mg/dL). Additional lab tests showed a carcinoembryonic antigen (CEA): < 1, cancer antigen 19–9 (CA 19–9): 4, and an elevated lipase: 584 (U/L).

Imaging studies performed at an external facility revealed a gastric outlet obstruction secondary to a large, multilocular cystic mass. The precise diagnosis of the mass remained unclear; however, a biopsy performed at an outside institution identified a spindle cell proliferation with low-grade epithelial proliferation, raising concern for low-grade pancreatic adenocarcinoma. Subsequently, the patient underwent an exploratory laparotomy to assess the extent of the mass. During surgery, the cystic mass was found adherent to the abdominal wall and ruptured intraoperatively upon dissection. The main tumor was also densely adherent to the left lateral section of the liver, gallbladder, colon just proximal to the splenic flexure, and antrum and pylorus of the stomach.

It appeared that the tumor arose from the pancreatic neck. It was decided then to perform an en bloc resection of the partial colon and stomach, gallbladder, distal pancreas, and spleen. Additional lymph nodes were sampled, and three liver wedge segments were taken to evaluate the spread of the tumor.

In follow-up, the patient was treated with gemcitabine/capecitabine, gemcitabine/abraxane, folinic acid (leucovorin) + fluorouracil (5-FU) + oxaliplatin (FOLFOX), and folinic acid (leucovorin) + fluorouracil (5-FU) + irinotecan (FOLFIRI) chemotherapies after surgery. However, the patient developed peritoneal metastasis a year after surgery and passed away after 2 years.

### Radiologic findings

**Fig. 1:**
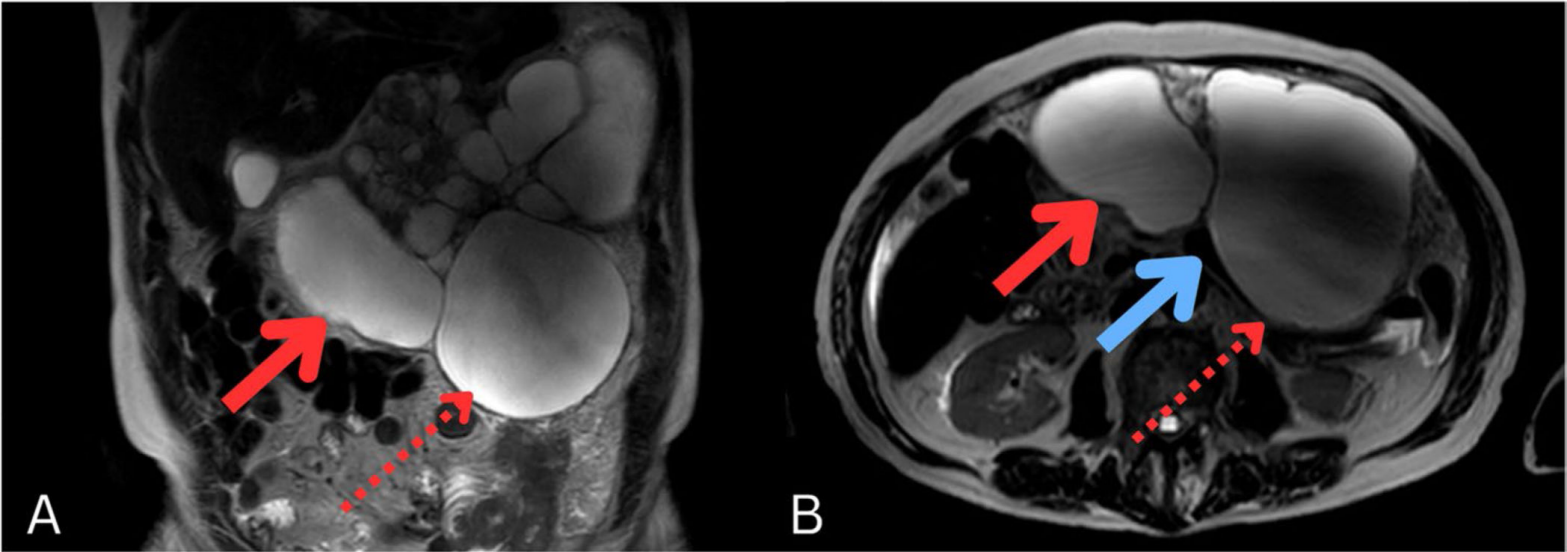
Preoperative MRI revealed **A**, **B** a large multiloculated cystic epigastric mass, measuring 21.2 cm × 12.6 cm × 18.6 cm, with two dominant cysts: red solid arrow points to smaller proximal cyst and red dotted arrow points to larger distal cyst. **B** The mass was contiguous with the pancreatic neck, causing pancreatic duct narrowing (blue solid arrow)

Abdominal magnetic resonance imaging (MRI) demonstrated gastric outlet obstruction secondary to a multiloculated cystic epigastric mass, measuring 21.2 cm × 12.6 cm × 18.6 cm, with two dominant cysts (Fig. 1A), which was an increase from 18.1 cm × 9.2 cm × 14.3 cm from 1 year prior. The mass remained contiguous with the pancreatic neck, causing pancreatic duct narrowing (Fig. 1B). Differential considerations included a peripancreatic cystic lesion, such as a lymphangioma with possible infection, or an uncommon pancreatic mucinous tumor extending into the peritoneal cavity. The mass exerted a significant mass effect, nearly encasing the gastric body. A separate 2.9 cm × 1.4 cm cystic lesion in the distal pancreatic tail remained stable, likely representing pancreatic duct ectasia. Recurrent peritoneal carcinomatosis was suspected. There is also a 0.5 cm cyst in hepatic segment 4A.

### Pathologic findings

**Fig. 2:**
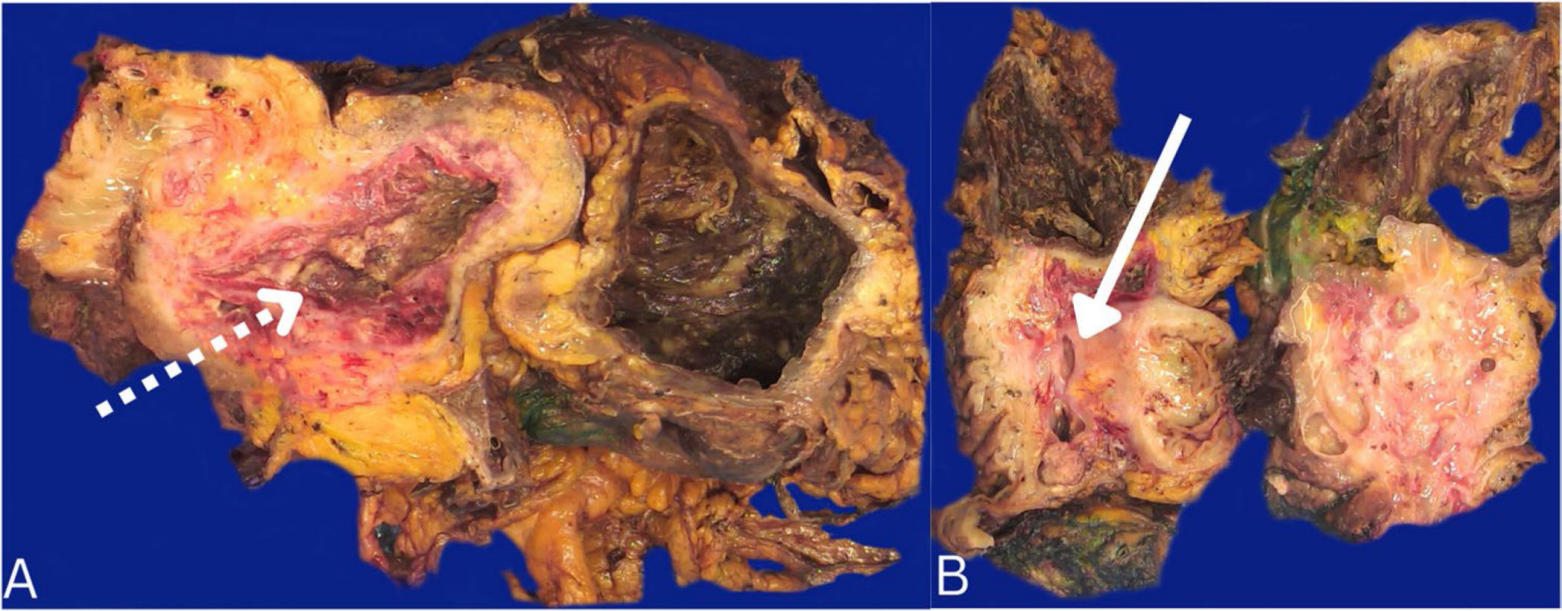
Gross examination of a large predominantly cystic mass in the distal pancreas. Sections revealed two dominant cysts. **A** The larger cyst located most distal to the pancreas had brown and nodular cyst lining (dotted white arrow). **B** The smaller cystic loculations were proximal to the larger cyst and contained tan to yellow purulent fluid and grumous material (solid white arrow)

Gross examination of the pancreas specimen revealed a large (25 cm × 22 cm × 9.5 cm) predominantly cystic mass that contains two dominant cysts. One larger distal cyst was devoid of contents, with the inner cyst lining being nodular and a wall thickness of up to 0.9 cm (Fig. 2A). The second smaller cyst proximal to the large cyst had both cystic and solid components, 40 and 60%, respectively (Fig. 2B). The solid component consisted of variably thickened tan fibrous septae with cystic loculations measuring up to 2.5 cm. These small cystic loculations contained predominantly tan/yellow thick purulent fluid and grumous material. Several loculations also contained prominent papillary excrescences (1.5 cm in greatest dimension). The segment 4 A liver wedge resection contained a 3.5 cm yellow-tan, multilobulated subcapsular mass with a yellow-tan, soft, and creamy cut surface.

**Fig. 3:**
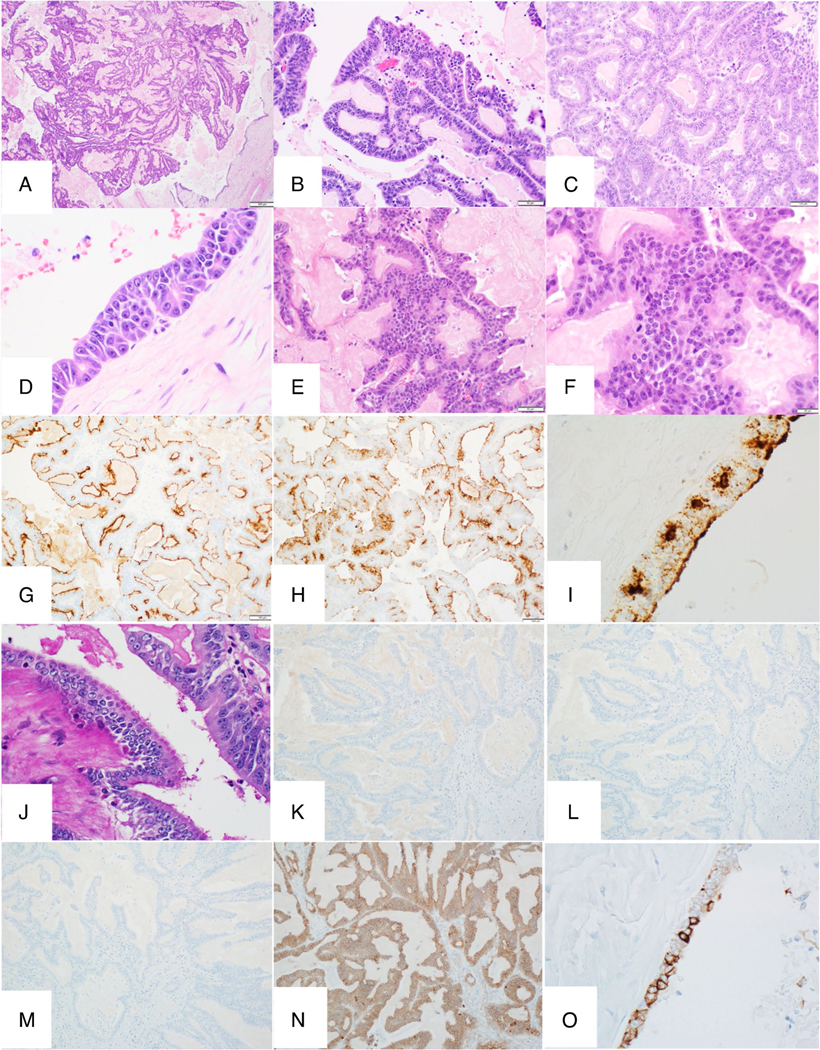
Histologic and immunohistochemical features of cystic and papillary variant of acinar cell carcinoma. **A** Low power view showing a well-circumscribed, cystic tumor with papillary proliferation of epithelial cells and eosinophilic proteinaceous luminal material. **B** Higher magnification reveals true fibrovascular cores lined by columnar cells with abundant eosinophilic cytoplasm, open chromatin, and prominent nucleoli. **C** Focal tubular architecture. **D** Areas of acinar differentiation with two-tone granular cytoplasm. **E**, **F** Liver metastasis shows similar cystic and papillary features. **G**–**I** Diffuse positivity for trypsin **G**, **I** and BCL-10 **H** confirms acinar differentiation in the tumor **G**, **H** and cyst lining **I**. **J** PAS stain shows granular cytoplasmic positivity. **K** AFP, **L** chromogranin A, and **M** synaptophysin are all negative. **N** Beta-catenin shows membranous staining. **O** CK19 is positive in cyst lining areas where trypsin/BCL-10 are mostly negative

Histologic evaluations at low magnification showed the tumor is well circumscribed and cystic with a predominantly papillary proliferation of epithelial cells (Fig. 3A). There was abundant eosinophilic amorphous proteinaceous material in the lumen. At higher magnification, true fibrovascular cores were present within the papillae (Fig. 3B). The papillary fronds were lined by columnar epithelial cells with abundant eosinophilic cytoplasm, enlarged uniformly round to oval nuclei with open chromatin, and a single prominent nucleolus. Mitotic figures were frequently observed. In focal areas, tubular architecture was also present (Fig. 3C). Some areas of the cyst lining consisted of epithelial cells with distinct acinar architecture and cytology characterized by abundant two-tone colored granular cytoplasm and a single prominent nucleolus (Fig. 3D), supportive of acinar differentiation. Other areas of the cyst lining consisted of cuboidal epithelial cells resembling ductal epithelial cells. No overt pleomorphism was identified in our case. The liver lesion showed similar cystic and papillary architecture and cytologic features with the pancreas lesion (Fig. 3E, F). Immunohistochemical staining demonstrated that the neoplastic cells showed diffuse positivity for the acinar markers trypsin (Fig. 3G) and BCL-10 (Fig. 3H), including some areas of cyst lining (Fig. 3I), thereby confirming acinar differentiation. Additionally, the neoplastic cells were positive for PAS (Fig. 3J), but negative for AFP, chromogranin, synaptophysin, and nuclear beta-catenin (Fig. 3K–N, respectively). CK19 was positive in cyst lining areas where trypsin/BCL-10 were negative (Fig. 3O), supporting ductal differentiation in those regions.

FoundationOne next-generation sequencing of the tumor showed mutations in NRAS, CTNNB1, and SMAD4 with microsatellite stability and low tumor mutation burden. There was no KRAS mutation. Together with the histologic findings, this tumor was classified as a cystic and papillary variant of acinar cell carcinoma with metastasis to the liver.

## Discussion

Metastatic cystic and papillary variants of pancreatic ACC to the liver maintaining the same morphology with more aggressive clinical behavior have not been previously reported. A summary of the differential diagnoses is listed in Table 1, adapted from the World Health Organization Classification of Tumours Editorial Board, 5th edition [13]. Cystic and papillary variants of ACC are extremely rare, with only a few reports in the literature [14–16]. This rare pattern of growth can be easily mistaken for other more common cystic and papillary neoplasms of the pancreas. The largest study of this variant by Basturk O. et al. included seven patients, and only one patient had liver metastasis, compared to 50% metastasis in classic ACCs [14]. This study implied that this variant may have a more indolent behavior compared to classic ACCs. There are four novel findings in our case report that have not been previously described and are worth reporting to the pathology community. First, we provided a description of the liver metastasis that histologically resembles the cystic and papillary architecture of the primary pancreas tumor. Second, we described somatic NRAS, CTNNB1, and SMAD4 mutations in our case. Third, we characterized the cyst lining with H&E and immunohistochemistry analysis, which demonstrated both acinar and ductal differentiation, suggesting that the tumor might be arising from benign acinar cystic transformation with acinar-to-ductal metaplasia instead of a true intraductal neoplasm. Finally, compared to the patient in the Basturk study who was alive after 28 months of progression with liver metastasis, this tumor is more aggressive, with the patient developing peritoneal metastasis 1 year after the surgery and subsequently passing away after 2 years, suggesting that some of these tumors might have a more aggressive behavior.

Histologically, ACCs are typically composed of either an acinar architecture, resembling normal pancreatic acini with small lumens, or a solid architecture, characterized by large sheets of cells without lumen. Less common patterns of ACC include the glandular pattern characterized by tubules with irregular lumens and trabecular pattern with ribbons of interconnecting plates. Both the trabecular and solid patterns can often be confused for pancreatic neuroendocrine tumors (PNETs) [1–5]. However, the distinct salt-and-pepper chromatin, inconspicuous nucleoli, and neuroendocrine markers synaptophysin and chromogranin expression make PNET distinguishable from ACC [1–5]. Acidophilic proteinaceous material is common in ACCs. Other rare variants of ACC include oncocytic, spindled cell, clear cell, and pleomorphic [1–5]. ACCs typically exhibit uniform neoplastic cells with a single prominent nucleolus, as in this case. However, we have seen many ACCs have inconspicuous nucleoli, emphasizing their morphologic diversity and the potential for atypical presentations. Therefore, careful evaluation of cytological and architectural features remains crucial in the initial assessment and differentiation of pancreatic neoplasms. Genetically, TP53, APC, SMAD4, SND1-BRAF, BRCA2, CDKN2A, and CTNNB1 mutations or fusions are some of the most frequently discovered alterations in ACC [17]. Microsatellite instability has also been identified in 8–14% of ACC [17]. However, the most common molecular alterations found in ductal adenocarcinoma (KRAS), cystic neoplasms (GNAS and RNF43), and NENs (MEN1, DAXX, and ATRX) are rarely found in ACC [1–4, 8, 12, 13, 17].

Distinguishing a cystic and papillary variant of ACC from IPMN among other cystic and papillary neoplasms of the pancreas can be challenging with limited sampling and in the absence of ancillary studies. IPMNs exhibit papillary features similar to those of papillary ACCs. However, IPMNs often demonstrate more abundant mucin and a lack of eosinophilic secretion. While the gastric and intestinal subtypes of IPMNs are easier to distinguish from ACCs morphologically due to their mucinous epithelium, caution should be exercised when differentiating a pancreatobiliary-type IPMN from ACC since they often display high-grade dysplasia and less cytoplasmic mucin [5, 6]. However, the presence of eosinophilic secretions and the abundant eosinophilic cytoplasm with a single prominent nucleolus provide additional clues suggestive of ACC. Identifying areas displaying more typical acinar morphology can also be helpful in making this diagnosis.

ITPNs represent another intraductal pancreatic neoplasm that can be confused with ACC, as ITPNs exhibit crowded tubulopapillary architecture that often lacks mucin with high-grade cytologic atypia. However, ITPNs also lack the eosinophilic granular cytoplasm and eosinophilic luminal secretions characteristic of ACC [8]. Immunohistochemical stains for acinar markers (such as trypsin, chymotrypsin, and BCL-10) can further confirm the morphologic impression of ACC and aid in the differentiation between these papillary neoplasms [1, 8].

Pancreatoblastoma is another rare tumor that can often be confused with ACC, as they often also present as a solitary lobulated cellular mass [12]. The acinar pattern predominates most of the tumor, while the ductal component is often very focal. A key distinction is the presence of squamoid nests that are almost always seen in pancreatoblastoma, but not ACC. Focal areas of neuroendocrine differentiation can be seen in these lesions and should not be confused with PNETs [11].

Pancreatic ductal adenocarcinoma (PDAC) is crucial to identify because the treatment differs significantly from that of ACC. Conventional PDAC can often be morphologically distinguished on H&E due to its small-caliber tubular structures with haphazard architecture and notable desmoplastic stromal reaction. However, a rare histologic variant of PDAC exists, described by Kelly et al., which presents as a cystic papillary pattern [9]. This variant can easily mimic other papillary or cystic lesions, such as IPMN. An important finding from Kelly’s study was that none of the nine cases exhibited a pure papillary cystic pattern. However, eight of the cases had a predominant papillary/cystic pattern of over 50% of the tumor [9]. The cysts and papillae in this variant were lined by epithelium with intracellular and intraluminal mucin, a key feature not observed in ACC. Elastin staining confirmed the invasive nature of these tumors, as there was a lack of elastin fibers around the tumor, unlike in normal ducts. Immunohistochemical stains show that this variant of PDAC stains like PDACs and is positive for MUC1 (EMA), MUC5AC, and MUC6 while negative for MUC2. This pattern of expression can be helpful for differentiation, as ACC often is negative for MUC1 and MUC2. Molecularly, these variants have no difference from conventional PDAC, with the study showing loss of SMAD4 [9].

Finally, the cystic and papillary variants of ACC also need to be differentiated from solid pseudopapillary neoplasms. These tumors are low-grade pancreatic neoplasms and of uncertain differentiation. They are characterized by nuclear beta-catenin expression. Architecturally, these tumors also present as a large solitary mass that can be encapsulated. Often, these tumors are cystic and contain friable material, mimicking a papillary appearance. However, fibrovascular cores are absent, and other clues of degeneration, such as macrophages, hemorrhage, and calcifications, are often present. Cytologically, these tumors often share uniform oval nuclear contours with ACC and can have eosinophilic cytoplasm. SPN, however, often has a characteristic longitudinal nuclear groove and inconspicuous nucleoli, differentiating it from ACC. In addition, the nuclei are often arranged away from the capillaries. Immunohistochemistry can be helpful to confirm your H&E impression, underscoring the value of ancillary studies in resolving diagnostic ambiguities, particularly in challenging cases and rare ACC variants. Besides nuclear beta-catenin, SPNs are positive for cyclin D1, CD10 (cytoplasmic), TFE3, and SOX11. It has been shown that synaptophysin can also be positive [10].

When evaluating a papillary or cystic lesion, it is important to consider a broad differential diagnosis as many entities can have this morphological appearance, including IPMN, ITPN, PDAC, and SPN. Key morphologic findings suggesting ACC include acinar architecture, prominent nucleoli, eosinophilic secretions, and abundant eosinophilic granular cytoplasm. Ancillary studies, particularly immunohistochemical stains, are paramount in diagnosing this entity, and molecular studies could possibly be helpful in difficult cases. We present a unique case of an acinar cell carcinoma with cystic and papillary patterns and liver metastasis. This paper aims to highlight the aggressiveness of this ACC variant, which has previously been suggested to be more indolent compared to conventional ACC. It is important to note that this pattern has been reported in only a few case reports, and more research is needed to understand its true prognosis and biology.

## Figures and Tables

**Table 1:** Differential diagnosis of cystic and papillary pancreatic neoplasms, including intraductal neoplasms, solid pseudopapillary neoplasm, and the papillary variant of pancreatic ductal adenocarcinoma (Adapted from WHO Classification of Tumours: Digestive System Tumours, 5th Edition, 2019) [13]

Tumor	Architectural features	Cytologic features	IHC	Molecular	Prognosis

ACC, cystic and papillary type	Abundant eosinophilic proteinaceous secretions in lumen. Cystic and papillary architecture	Eosinophilic cytoplasm with round to oval nuclei and single prominent nucleoli. Two tone colored granular cytoplasm	BCL-10, trypsin, chymotrypsin	TP53, APC, SMAD4, SND1-BRAF, BRCA2, CDKN2A, CTNNB1 mutations or fusions. MSI also seen	Poor prognosis with a median survival time of 19 months
Ductal adenocarcinoma	Duct-like glandular structures that haphazardly infiltrate the parenchyma. Desmoplastic stromal response	Variable, can be well differentiated with bland ducts to poorly differentiated carcinomas with various subtypes	No specific IHC marker. CK7+, CK19+. Usually EMA+ and MUC5AC+. Typically negative for neuroendocrine markers and acinar markers	KRAS, loss of SMAD4, TP53, and CDKN2A (P16)	Fatal in almost all cases. Mean untreated survival time is 3–5 months and 10–20 months after surgical resection
IPMN	Papillary features similar to papillary ACCs. Lack eosinophilic secretions	Abundant mucin. Less eosinophilic cytoplasm compared to ACC	CK7+, CK19+. CDX2+ (intestinal subtype), CK20+ (intestinal subtype)	No clinically relevant molecular findings	Often curable if without invasive carcinoma. 5-year survival rate after resection is 100% if low grade and 85–95% if high-grade
ITPN	Tubulopapillary, cribriform architecture. Lack eosinophilic luminal secretions	High grade cytologic atypia and lack mucin. Lack eosinophilic granular cytoplasm	CK7+, CK19+,EMA+, MUC6+, CK20-, acinar markers-, neuroendocrine markers-, MUC2-	No clinically relevant molecular findings	Significantly better than ductal adenocarcinoma. 5-year survival rate is 71% in ITPNs with invasive carcinoma
Pancreatoblastoma	Predominantly acinar pattern, focal ductal component, squamoid nests critical for diagnosis	Single prominent nucleolus with modest nuclear atypia. Squamoid nests lack atypia, prominent nucleoli, and mitoses	Acinar component stains trypsin+, chymotrypsin+, and BCL-10+. AFP+, nuclear beta-cateinin	Loss of heterozygosity of short arm of chromosome 11p. Alterations in APC/beta-catenin pathway	Indolent and curable tumor but can be locally invasive and recurrent. Occasional distant metastasis
SPN	True fibrovascular cores are absent. Friable tumor	Round to oval nuclei, nuclear grooves. Finely dispersed chromatin without prominent nucleolus. Mitoses are uncommon	Nuclear/cytoplasmic expression of beta-catenin. CyclinD1+, CD10+ (cytoplasmic), TFE3+, SOX11 +	Activating mutation in exon 3 of CTNNB1	Excellent long-term disease-free with long disease-free periods after complete surgical resection. Currently classified as a low-grade malignant neoplasm

## Data Availability

The datasets generated and/or analyzed during this study are available from the corresponding author on reasonable request.

## Abbreviations

ACC: Acinar cell carcinoma
SPN: Solid pseudopapillary neoplasm
PNET: Pancreatic neuroendocrine tumor
PB: Pancreatoblastoma
IPMN: Intraductal papillary mucinous neoplasm
ITPN: Intraductal tubulopapillary neoplasm
